# Effects of moderate high temperature and UV-B on accumulation of withanolides and relative expression of the squalene synthase gene in Physalis peruviana

**DOI:** 10.3906/biy-2002-69

**Published:** 2020-10-13

**Authors:** Günce ŞAHİN, Murat TELLİ, Ercan Selçuk ÜNLÜ, Fatma PEHLİVAN KARAKAŞ

**Affiliations:** 1 Department of Biology, Faculty of Arts and Science, Bolu Abant İzzet Baysal University, Bolu Turkey; 2 Department of Chemisty, Faculty of Arts and Science, Bolu Abant İzzet Baysal University, Bolu Turkey

**Keywords:** Cape gooseberry, withanolides, squalene synthase, HPLC, qPCR1

## Abstract

*Physalis peruviana* L. (Cape gooseberry) is a source for a variety of phytocompounds such as withanolides, withanone, withaferin A, and withanolide A. These withanolides are high-value drug candidates due to their various pharmacological properties. To meet the increasing demands for these compounds, plant cell technology offers a reliable alternative. Exogenous addition of elicitors is considered the most effective strategy for enhanced production of secondary metabolites. In this study, we investigated changes in withanolide accumulation and characterized the gene expression level changes of squalene synthase enzyme in *P. peruviana* shoot cultures exposed to mild nonlethal heat stress (45°C for 2 and 5 h) and UV-B radiation (313 nm for 15 min and 3 h). We demonstrated significant changes in withanolide content with 7.86- and 12.5-fold increases for 2- and 5-hmild high-temperature exposure times, respectively. Exposure to UV-B also changed the withanolide content by 7.22- and 7-fold increases for 15 min and 3 h exposure times, respectively. The relative expression level of squalene synthase gene showed consistent results with1.80- and 10.13-fold increases in withanolide for 2- and 5-h mild high-temperature exposure times, and 1.34- and 2.01-fold increases with 15 min and 3 h UV-B exposure times, respectively.

## 1. Introduction

*Physalis peruviana* L., Cape gooseberry, belonging to the family Solanaceae and genus *Physalis* , is a source of a variety of compounds with potential health benefits such as minerals, withanolides, physalins, carotenoids, provitamin A, and cinnamic acid-derived volatiles.  Leaves, stems, and fruits of the plant have been widely used as a sedative and as an analgesic, and to treat several diseases including diabetes, hepatitis, ulcers, and dermatitis. Withanolides are complex steroidal lactones that exhibit significant pharmacological activities including hepatoprotective activity against CCl4-induced hepatoxicity, as well as antibacterial, antiinflammatory, antitumor, insect repellent, immunomodulatory, and cytotoxic activities (Glotter, 1991; Mishra et al., 2000; Grover et al., 2013). Medicinal clinicians and chemists are interested in the chemical synthesis of beneficial withanolides such as withanone, withaferin A, withanolide A, and withanolide D. However, the synthetic production of these substances is challenging due to the presence of chiral centers, a high energy epoxy ring, a stereochemical structure, and rigid translactone groups. Thus, synthetic production of withanolides is economically impractical because of the high production cost. On the other hand, in vitro culture of *Physalis* sp., which is more susceptible to environmental conditions, has other problems because of its low growth rate, long germination time, and complex accumulation pattern. Withanolides are primarily localized in leaf tissues, but can be found in different vegetative plant tissues in varying amounts (from 0.001% to 0.5% of dry weight) (Grover et al., 2013). 

Development of cost-effective techniques to produce withanolides is required. Improving in vitro culture methods is a promising approach. Exogenous elicitor application is the most effective strategy for enhanced production of secondary metabolites (Radman et al., 2003). Several reports have demonstrated that the application of abiotic agents such as heat, drought, salt, UV radiation, and water stress increase the accumulation of these metabolites. These elicitors act as intracellular or extracellular signals. The membrane receptor perceiving the signal can initiate a signaling cascade that is required for the de novo biosynthesis or activation of transcription factors that regulate the expression of genes involved in secondary metabolism (Zhao et al., 2005; Kim et al., 2009). Various environmental stressors such as heat and/or UV-B radiation strongly influence metabolic activity, and can stimulate levels of metabolites such as ascorbic acid, phenolic contents, carotenoids, etc. Mechanisms can be related to the upregulation of defense-related secondary metabolism, i.e. phenylpropanoids and the isoprenoids pathway, resultingin the rise of phenolic compound concentrations, alkaloids, and terpenoid production (Gerhardt et al., 2008). 

Severe abiotic stresses at high levels result in detrimental consequences to plant growth. However, mild stress conditions may be deliberately applied to improve the content of antioxidants and secondary metabolites in the edible parts of the plant and stimulate plant adaptation to stress conditions (Galli et al., 2016). Plants can acquire thermotolerance when exposed to mild high temperatures for a long time or sublethal high temperatures for a short time (Sung et al., 2003). Moderate UV-B radiation is known to cause distinct changes in the plant’s secondary metabolism and upregulate genes encoding enzymes of the phenylpropanoid pathway (Jansen et al., 2008; Schreiner et al., 2009; Zhang and Björn, 2009; Obata and Fernie, 2012). In other words, although very high or low stress intensity imposes different metabolic and physical challenges, mild stress conditions for acquired tolerance cause the accumulation of stress-specific components as well as plants’ specific metabolites (Galli et al., 2016).

This study investigated the impact of moderate stress-inducing UV-B and temperature applications on the dynamics of health-promoting plant metabolites of *Physalis peruviana* L. Information on different treatment conditions of UV-B light intensity and heat application on phytochemical production will contribute to the selection of the most appropriate environment for growing medicinal plants with enhanced phytochemical concentrations. The data will be applicable for further commercialization and exploration of novel methods on a pilot scale in pharmaceutical industries.

The biosynthesis pathway of withanolides is dependent on the flux of isoprenoid (C5), which is then directed into triterpene and sterol biosynthesis (Chaurasiya et al., 2012). The enzyme of squalene synthase (SQS: EC 2.5.1.21) is located at the divergent branch point of the isoprenoid pathway to sterol biosynthesis. Studies with mutant strains of squalene synthase have shown its regulatory function on sterol biosynthesis in yeast cells (Tozawa et al., 1999). Metabolic manipulations to overexpress squalene synthase enzyme using transformed cell lines have resulted in an increased level of withanolide contents in *Withaniasomnifera* (Grover et al., 2013). Thus, the squalene synthase gene is considered to bea central regulatory enzyme for the withanolide biosynthesis pathway. However, the biosynthetic pathway of withanolides is not fully characterized. Squalene synthase encoding genes have been cloned and characterized for several plant species (Uchida et al., 2009) such as *Euphorbia tirucalli* , *Withaniasomnifera* , *Nicotiana tabacum* , *Glycyrrhiza glabra* , and *Panax ginseng* . These studies showed a change in expression of the SQS gene by various elicitor treatments, and the expression pattern corroborated positively with withanolide accumulation. However, there are no reports on *Physalis* cultures in which either improvement of withanolide production with specific elicitors or the genes related to withanolide synthesis have been investigated. 

Therefore, the aims of the present study were the following: (i) find the influence of abiotic elicitors (mild high temperature and UVB radiation) on the plants and withanolides such as withaferin A, withanone, and withanolide A; (ii) set up elicitation strategies to synthesize withanolides in higher amounts; and (iii) reveal expression differentiation of the squalene synthase gene under the effects of those UV and temperature elicitors. 

## 2. Materials and methods

### 2.1. Plant materials and experimental design

Seeds of *P. peruviana* were purchased from commercial sources in 2015 in Bolu, Turkey. Surface sterilization and germination procedures of seeds were performed according to a protocol previously published by Yücesan et al. (2015). Nodal segments isolated from the seedlings were cultured on MS medium containing 3% sucrose, 0.8% agar, and 0.5 mg L−l TDZ for direct shoot induction for 30 days. Maintenance of cultures was done by subculture every 2 weeks with replacement of the medium with the same composition. Thirty days later, cultures were elicited with 45 °C for 2 and 5 h to create mild high-temperature stress (Gulen and Eris, 2004). Cultures were exposed to varying periods (15 min and 3h) of UV-B light by placing the plant culture vessels on a UV illuminator with 313-nm wavelength to create UV-B elicitation (Takshak and Agrawal, 2014). For the control group (T_0_), cultures were grown in a culture room with a controlled environment (23 ± 2 °C; 16-h light: 8-h dark photoperiod). Leaves from both cultures (treated and control) were stored at −80 °C until total RNA isolation and high-performance liquid chromatography (HPLC) analyses. For each treatment, 12replicate vessels were maintained.

In the present experiment, different treatment conditions are as follows: 

UV1: UV-B exposure; PAR + UV-B for 15 min treatment (T_15m_);

UV2: UV-B exposure; PAR + UV-B for 3h treatment (T_3h_);

TEMP1: Temperature exposure; 45 °C for 2 h (T_2h_);

TEMP2: Temperature exposure; 45 °C for 5 h (T_5h_);

Control: 23 ± 2 °C, PAR and UV-B excluded.

### 2.2. Extraction of withanolides and HPLC analysis

Withanolide extraction and the analytical HPLC experiments were determined according to a protocol previously published by Takshak and Agrawal (2014). Five additional calibration levels of each standard compound (withanolide A, withanolide, and withaferin A) were prepared by diluting the stock solutions with concentrations ranging from 0 to 100 ppm. Separation of withanolides was performed by HPLC; 10 µL of each sample was injected using a WPS-3000-SL Dionex Semi Prep Autosampler (Dionex Corporation, Sunnyvale, CA, USA). All sampling was carried out with a UV-DAD detector (MWD-3100 Dionex UV-VIS Detector) operating at 229 nm, with a flow rate of 0.6 mL/min (binary pump, LPG 3400SD Dionex) at 30 °C (column oven system, TCC3000SD, Dionex) and an Inertsil ODS-3 (GL Sciences Inc., Tokyo, Japan) column (150 × 4.6 mm). The isocratic flow was performed using methanol/water containing 1% trifluoroacetic acid (w/v) in a gradient fashion (45:55–65:35) for 15 min (Grover et al., 2013).

### 2.3. Total RNA isolation and cDNA synthesis

Fifty mg of leaf tissue of *P. peruviana* from 3 different biological replicates was homogenized using nuclease-free bead-beating tubes. Homogenates were then preceded immediately to total RNA extraction using the TRIZOL method (Invitrogen Corporation, Carlsbad, CA, USA). Total RNA extracts were treated with TURBOTM (Ambion, Austin, TX, USA) DNase treatments and removal reagents to prevent potential DNA contamination during total RNA extraction.  RNA integrity and quantity were checked using 1.5% denaturized agarose gel and spectrophotometry. Single-stranded cDNA syntheses were carried out using a Thermo RevertAid First Strand cDNA Synthesis Kit according to the manufacturer’s instructions. cDNAs were synthesized with around 1.5 µg total RNA and stored at –80 °C.

### 2.4. Real-time PCR analysis

18S rRNA was used as the endogenous control gene of *P. peruviana.* Squalene synthase (also known as farnesyl–diphosphate or farnesyl transferase) was used as the target gene in RT-PCR experiments. The nucleotide sequence of 18SrRNA (control gene ID: Php00a08602.18555) and squalene synthase (target gene ID: Php00a07480.17433) were parsed from the leaf transcriptome database of *P. peruviana* published by Martinez et al. (2012) using custom-built Perl codes. 

RT-PCR was conducted on a CFX-96 Touch Bio-Rad real-time PCR system (Bio-Rad Laboratories, Inc., Philadelphia, PA, USA) for 3 biological replicates. Each reaction contained 10 ng cDNA template, 10 µL iTaq Universal SYBR Green Supermix (Bio-Rad Laboratories, Inc.), and 300 nM of each primer in a final volume of 20 µL. For the amplification of squalene synthase, we used 5’-GCCTAAGTACAGTCCCATTC-3’ (forward) and 5’-CTTCCTAGACCGAACTTCTC-3’ (reverse) primers. The amplification of the 18S rRNA control gene was carried out using 5’-CTCTCATCCAGACCATCAAG-3’ (forward), 5’-CTCGATAGGGCTGAAAAGG-3’primers. Each reaction was conducted in triplicate and a nontemplate negative control and nonreverse transcriptions were included. Cycling parameters were 95 °C for 30 s to activate the DNA polymerase, followed by 40 cycles of 95 °C for 5 s and 58 °C for 30 s. The primer specificity was assessed by melting curve analyses between 45–95 °C by 0.5 °C increments at 2 s per step. PCR amplification efficiency was measured in triplicate by a standard curve generated using a 2-fold dilution series. The threshold cycle (Ct) values were normalized against the control gene of 18S rRNA. The relative expression levels were analyzed using the ΔΔCt method (Livak and Schmittgen, 2001) and represented as fold changes in gene expression relative to the initial time of treatments (T_0_ h). 

### 2.5. Statistical analysis 

All of the experiments were repeated thrice under identical conditions for statistical reliability. A one-way ANOVA (analysis of variance) was applied to confirm the significance of the data according to Duncan’s multiple range test at P ≤ 0.05 using SPSS16.0 software.

## 3. Results and discussion

**Figure 1:**
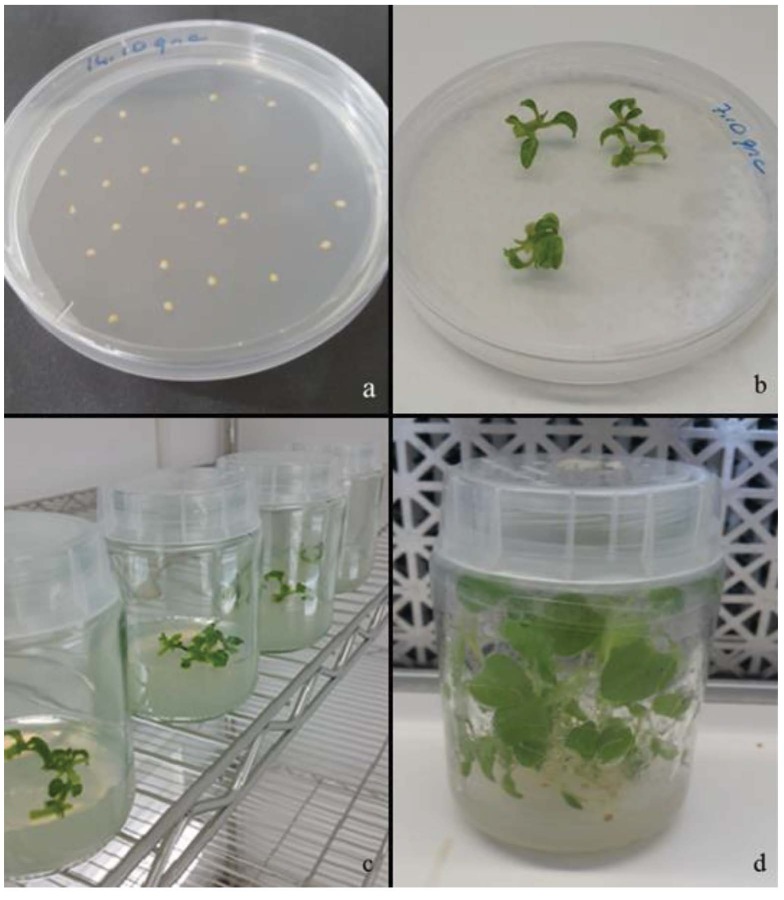
In vitro shoot regeneration from nodal explants of P. peruviana. a) Seeds on the germination medium (MS), b) Shoot induction on MS medium containing 0.5 mg L^−l^ TDZ, c–d) Plantlets gradually formed many shoots after subculture on the same medium.

**Figure 2:**
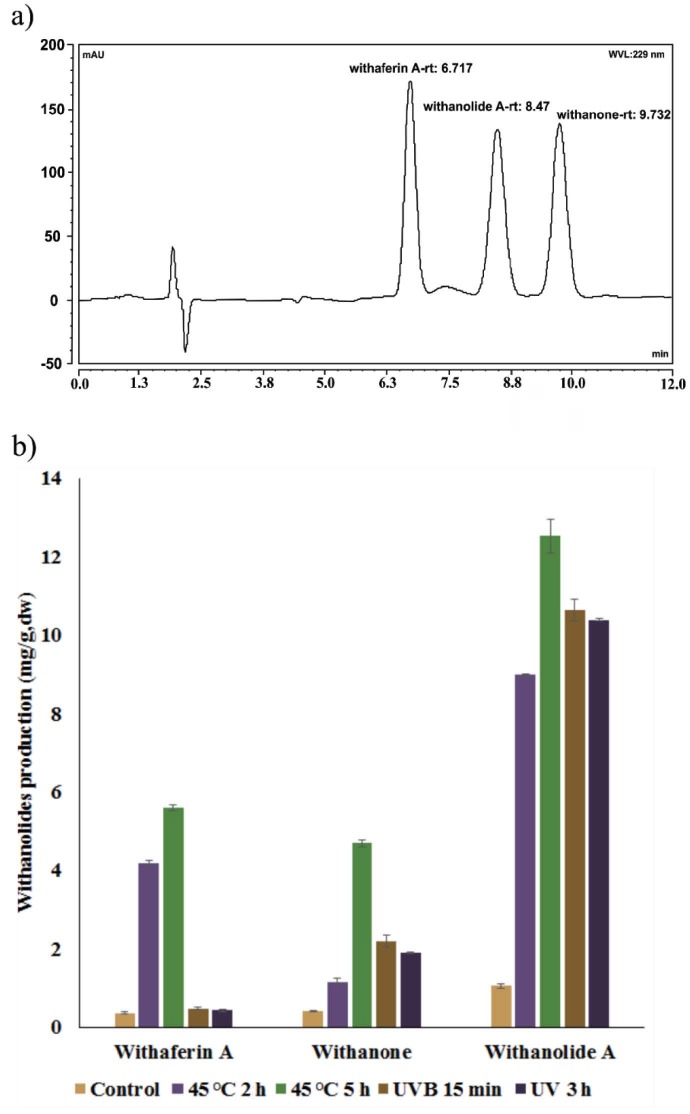
HPLC analysis of methanol extracts from shoot cultures of P. peruviana. a) Standards of withaferin A, withanolide A and withanone, b) Comparison of withanolide contents.

In this study, we aimed to determine changes in withanolide accumulation and squalene synthase gene expression levels in *P. peruviana* shoot cultures (Figure 1) exposed to mild high temperature and UV-B radiation. We were able to obtain enhanced production of withanolides from regenerated shoots derived from the nodal segments with the elicitation of mild high temperature and UV-B irradiation. We analyzed withanolide accumulation by HPCL analysis. HPCL results for standard samples are presented in Figure 2a. Elicitor treatment promoted the total withanolide accumulation in leaves (Figure 2b). The high degree of variation in the quality and quantity of active principles in different organs has been reported in previous studies for different plant species (Kumar et al., 2007; Kumar et al., 2011; Kitchlu et al., 2011; Cirak et al., 2012; Katoch et al., 2012). Changing amounts of withanolide accumulation in different plant tissues of *W. somnifera* were previously reported by Kumar et al. (2007). It was also found that leaves are rich in withaferin A content, while withanolide A is mostly accumulated in the roots (Mirjalili et al., 2009; Mir et al., 2013). Therefore, in our study, shoots were obtained from nodal segments after 30 days of culture (Figure 1). Yücesan et al., (2015) induced maximum shoot production from internode explants of in vitro grown plants of *P. peruviana* on MS medium containing 0.5 mg L−l TDZ. Thus, in the present study, the same hormonal concentration was selected for maximum shoot induction from the nodal explants.

The culture turned brown after 40 days. It has been suggested that culture browning could be due to either phenolic compounds or loss of viability of the cultures (Zhao et al., 2005). In the present study, 30-day-old shoot culture was found to be an optimal culture age for elicitor treatment. 

Optimization of the elicitation period is one of the critical factors for improving the secondary metabolite production in plant cell and tissue cultures. Thirty-day-old cultures were exposed to moderate high-temperature stress using 45°C for 2–5 h and UV-B radiation using 313 nm for 15 min/3 h to enhance withanolide production in *P. peruviana* shoot cultures, after considering the maximum accumulation of plant steroids in several plant species with minimal loss of cell viability based on the literature data (Weathers et al., 1990; Kumari and Prasad, 2013). Among all of the treatments, the most widespread withanolide was withanolide A in the leaves, which ranged between 1.060 mg.g–1and 12.51 mg.g–1 (Table).

**Table:** Table. Analysis of three different withanolides in the control (nontreated) and elicited cultures of Physalis peruviana. Data are means ± SD from three replicates. Values followed by different letters in the same column are significantly different (P < 0.05).

	Amounts of withanolides (mg g-1dw)			
Types of elicitors	Withaferin A	Withanone	Withanolide A	Total
Control	0.35j ± 0.036	0.41j ± 0.021	1.06i ± 0.05	1.82
45 °C 2h	4.17f ± 0.08	1.16i ± 0.084	8.97c ± 0.023	14.3
45 °C 5h	5.58d ± 0.08	4.68e ± 0.1	12.51a ± 0.43	22.77
UVB 15 min	0.47j ± 0.02	2.17g ± 0.15	10.63b ± 0.27	13.27
UV 3 h	0.42j ± 0.02	1.89h ± 0.01	10.38b ± 0.02	12.69

A remarkable increase in withanolide A (12.51 mg.g–1dw), withaferin A (5.58 mg.g–1dw), and withanone (4.68 mg.g–1dw) content was prominent in 5-h mild high-temperature–treated cultures when compared with the concentrations of withanolide A (1.06 mg.g–1dw), withaferin A (0.35 mg.g–1dw), and withanone (0.41 mg.g–1dw) in the control groups (Figure 2b). In addition, 2 h mild high-temperature exposure was also very effective in increasing production of withanolides. Cultures elicited with UV-B (15 min or 3h) showed significantly increased levels of withanolide A and withanone contents. However, withaferin A content was not significantly changed compared to control groups after UV-B exposure. It was reported by Neugart et al. (2014) that monoacylated quercetin glycosides were increased by mild high temperature and UV-B in *Brassica oleracea.* Many plants have been shown to increase their alkaloid content under UV-B treatment. For instance, UV light-induced the formation of terpenoid indole alkaloids as well as their precursors in *Catharanthus roseus* (Binder et al., 2009). Moderate UV-B radiation is known to upregulate genes encoding enzymes of the phenylpropanoid pathway (Jenkins, 2009). Only a few publications describe targeted low or moderate UV-B radiation treatments to increase the level of flavonoids (Morales et al., 2010; Lancaster et al., 2000).

In the present study, we also investigated the effect of overexpression of squalene synthase by various elicitor treatments in shoot cultures of *P. peruviana* for production of withanolides. Squalene synthase is a potential branch point regulatory enzyme and represents the first committed step to diverging the carbon flux from the main isoprenoid pathway towards sterol biosynthesis. Many approaches have been investigated to understand the regulatory role of SQS in sterol biosynthesis using specific inhibitors of SQS (Wentzinger et al., 2002), SQS mutants (Tozawa et al., 1999) and fungal elicitors (Vogeli and Chappell, 1988). It has been reported that overexpression of SQS genes led to the enhanced accumulation of steroidal compounds, phytosterols, and/or triterpenes in *Withania somnifera* (Patel et al., 2015), *Withania coagulans* (Mirjalili et al., 2011), *Bupleurum falcatum* ( Kim et al., 2011), *Eleutherococcus senticosus* (Seo et al., 2005), and *Panax ginseng* (Lee et al., 2004).

**Figure 3:**
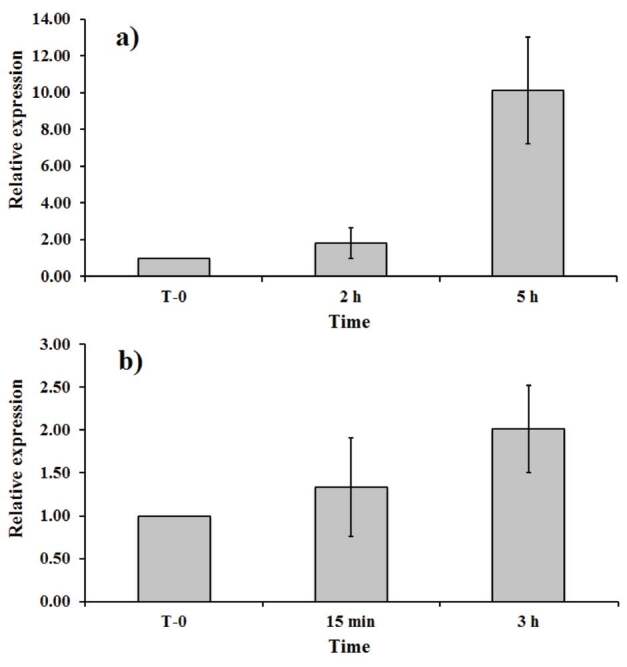
The relative expression levels of squalene synthase gene represented as fold changes in gene expression relative to the initial time of treatments (T-0 h) in temperature (a) and UV-B (b) stress conditions.

In our study, RT-PCR results showed significantly higher expression of the SQS gene relative to T_0_ in 2- and 5-h exposure time of temperature in *P. peruviana* (Figure 3). Although there is a wide range of expression levels of biological replicates of T5h (8- to 12-fold), the differences between T2h and T5h are statistically significant (P < 0.001). Our results show that the increase in the relative expression level of the SQS gene is time-dependent. The exposure time affecting variations in the SQS gene expression for the plant samples treated with mild high temperature is consistent with the accumulation of withanolides (Figure 3). The exposure times of 15 min and 3 h of UV did not show statistically significant differentiation in the relative expression of the SQS gene, although there is significant differentiation in their expression levels relative to T_0_. Data suggest that there is a relationship between increased levels of SQS and secondary metabolite production. The impact of temperature and UV may trigger overlapping and independent pathways to trigger the biosynthesis of various withanolides, as the types and levels of metabolites tested in the study show variations for the plants exposed to temperature or UV. 

Our results showed a significant exposure time dependent enhancement for the biosynthesis of withaferin A, withanone, and withanolide A under mild high-temperature conditions in *P. peruviana* . A similar trend in differential expression of the squalene synthase gene addresses its regulatory function for triterpene biosynthesis for *P. peruviana* . UV exposure-time dependency seems not to be the case for either withanolide biosynthesis or differential expression of the squalene synthase gene, although there is a significant difference relative to initial time (T_0_).

The elevated temperature might have several consequences on metabolic regulations such as permeability, transpiration, and water usage that might alter the physiology and metabolism of plant cell culture. Therefore, temperature variation alone might act as a multistress circumstance, changing the accumulation of secondary plant metabolites (Selmar and Kleinwächter, 2013). Effects of high temperature on secondary metabolites have shown inconsistent results depending on types of metabolites, species, and exposure times. Elevated temperature leads to increase leaf senescence and reduced photosynthesis rate, but increased ginsenoside accumulation in *Panax* *quinquefolius* (Akula and Ravishankar, 2011). However secondary metabolite accumulation is generally favored with lower temperatures in different species accompanied by upregulation of related metabolic gene expression (Thimmaraju et al., 2003). Inconsistent results concerning high-temperature effects on secondary metabolite accumulation might be closely related to exposure duration. Long-term chronic exposure for days may result in the death of the organism, whereas short-term acute exposure has varying results. We showed a significant and exposure-time–dependent response to short-term mild high-temperature treatment with a more than 12-fold increase of withaferin A, withanone, and withanolide A biosynthesis, and a more than 10-fold increase of squalene synthase gene expression in *P. peruviana* . Treatments of 45 °C for 2 and 5 h are likely not high enough to damage the metabolic processes of *P. peruviana* and therefore may enhance and increase the accumulation of secondary metabolites. This result proved that short-term mild high-temperature application as an elicitor was a particularly useful alternative method for promoting secondary metabolites for commercial biotechnology applications.

UV radiation is a well-documented stress factor mediating changes in the production of plant secondary metabolites such as terpenoids, alkaloids, glucosinolates, and tocopherols acting as antioxidants to reactive oxygen species (ROS) (Schreiner et al., 2012). Using low, ecologically relevant UV-B levels is widely considered a new opportunity in the agricultural industry to enhance the accumulation of antioxidant secondary metabolites in crop products (Kumari and Agrawal, 2011; Eichholz et al., 2011). Long-term and high doses of UV exposure result in decreasing terpenoid accumulation (Afreen et al., 2005) and inhibition of expression of related glucosinolate biosynthetic genes (Wang et al., 2011). However, there has been no comprehensive study revealing UV-mediated accumulation patterns of different secondary metabolites associated with exposure time and expression differentiation of related genes in the literature. In the present study, 15 min short-term moderate UV-B enhances the biosynthesis of withaferin A, withanone, and withanolide A, although an extended exposure time of 3 h did not change accumulation levels of those secondary metabolites. It seems that biosynthesis of terpenoids as phytoactive metabolites can be quickly induced by moderate UV-B application.    

## 5. Conclusion 

In conclusion, this is the first study aimed to enhance withanolide biosynthesis levels through considering the differential expression of the squalene synthase gene in *P. peruviana* . The results of the study show that exogenous addition of short-term mild high temperature and UV-Belicitors results in a significant enhancement in squalene synthase gene activity parallel with withanolide accumulation in *P. peruviana.* Therefore, those elicitors can be used as a biotechnological improvement of *P. peruviana* to increase withanolide biosynthesis for commercial biotechnology applications. 

**Acknowledgments**

This study was supported by the Bolu Abant İzzet Baysal University Research Foundation (Project No: BAP – 2016.03.01.994). 
